# A Prospective Cohort Study of Alcohol Exposure in Early and Late Pregnancy within an Urban Population in Ireland

**DOI:** 10.3390/ijerph110202049

**Published:** 2014-02-17

**Authors:** Deirdre J. Murphy, Clare Dunney, Aoife Mullally, Nita Adnan, Tom Fahey, Joe Barry

**Affiliations:** 1 Academic Department of Obstetrics and Gynaecology, Coombe Women and Infants University Hospital and Trinity College Dublin, Dublin 8, Ireland; E-Mails: cdunney@coombe.ie (C.D.); amullally@coombe.ie (A.M.);; 2 Coombe Women & Infants University Hospital, Dublin 8, Ireland; E-Mail: nita_adnan@hotmail.com; 3 HRB Centre for Primary Care Research, Department of Family Medicine and General Practice, Royal College of Surgeons in Ireland, Dublin 2, Ireland; E-Mail: Tomfahey@rcsi.ie; 4 Department of Public Health and Primary Care, Trinity College Dublin, Dublin 2, Ireland; E-Mail: Joebarry@tcd.ie

**Keywords:** alcohol exposure, prospective cohort study, pregnancy, perinatal outcomes

## Abstract

Most studies of alcohol consumption in pregnancy have looked at one time point only, often relying on recall. The aim of this longitudinal study was to determine whether alcohol consumption changes in early and late pregnancy and whether this affects perinatal outcomes. We performed a prospective cohort study, conducted from November 2010 to December 2011 at a teaching hospital in the Republic of Ireland. Of the 907 women with a singleton pregnancy who booked for antenatal care and delivered at the hospital, 185 (20%) abstained from alcohol in the first trimester but drank in the third trimester, 105 (12%) consumed alcohol in the first and third trimesters, and the remaining 617 (68%) consumed no alcohol in pregnancy. Factors associated with continuing to drink in pregnancy included older maternal age (30–39 years), Irish nationality, private healthcare, smoking, and a history of illicit drug use. Compared to pre-pregnancy, alcohol consumption in pregnancy was markedly reduced, with the majority of drinkers consuming ≤ 5 units per week (92% in first trimester, 72–75% in third trimester). Perhaps because of this, perinatal outcomes were similar for non-drinkers, women who abstained from alcohol in the first trimester, and women who drank in the first and third trimester of pregnancy. Most women moderate their alcohol consumption in pregnancy, especially in the first trimester, and have perinatal outcomes similar to those who abstain.

## 1. Introduction

Many women consume alcohol in pregnancy despite concerns that there may be risks to the developing fetus [1,2,3]. Moderate to heavy alcohol consumption has been associated with a number of adverse perinatal outcomes including preterm birth, low birth weight and intrauterine growth restriction [3,4,5,6]. However, results are inconsistent for low-level alcohol consumption [7,8]. Binge-drinking is an increasingly common behavior among women of reproductive age and is likely to be associated with unplanned pregnancy, raising further concern about inadvertent high level alcohol exposure in early pregnancy [9,10].

The Department of Health in Ireland advises that alcohol should be avoided in pregnancy. The Surgeon General in The United States goes further advising avoidance of alcohol both in pregnancy and when planning a pregnancy. However, the National Institute for Health and Clinical Excellence (NICE) in the United Kingdom take a more tolerant approach and recommends that women who want to drink in pregnancy should be advised to drink no more than one to two units once or twice a week [11]. In a previous Irish study we reported that 81% of women drink alcohol in the peri-conceptional period and that high alcohol intake (>20 units) is associated with very preterm birth [3]. In a further study, we reported an increased risk of IUGR among women who continued to drink at the time of the first booking visit compared to non-drinkers but this was mainly attributed to co-existing smoking [12].

The aim of this study was to recruit a prospective cohort of women booking for antenatal care and delivery in a university teaching hospital in Dublin to investigate their reported behavior in relation to alcohol consumption pre-pregnancy, at the time of the first antenatal visit, and in the third trimester of pregnancy, and the implications for perinatal outcomes. This study was part of a larger study addressing “Lifestyle Behaviours in Pregnancy” [13].

## 2. Methods

### 2.1. Sample and Recruitment

A prospective cohort study was carried out including women who booked for antenatal care and delivered in a large Dublin maternity hospital between November 2010 and December 2011. Women were eligible to be included if they had a singleton pregnancy, were aged 18 years or above and understood English. We would like to have recruited all women booking at the hospital but this was not possible for logistical and resource reasons. Given the wide range of settings for booking visits a pragmatic approach was used by research staff (a midwife and trainee obstetrician) to recruit from settings that had the greatest numbers of women booking on a given day. A sample size of 1,000 women was chosen based on the predicted difference in growth restricted babies for moderate and heavy drinkers compared to non-drinkers as reported in a previous study from the same hospital setting [12]. The initial sample size of 1,000 participants was inflated to 1,300 when a lower response rate to the third trimester questionnaire became apparent. Information leaflets were distributed to all eligible women and women interested in participating gave written consent.

### 2.2. Data Collection

Detailed information on lifestyle behaviours included consumption of alcohol, smoking, diet, exercise and infant feeding intention. Women were asked about their awareness of the units of alcohol in drinks and whether they were aware of the recommended number of alcohol units women should not exceed. They were asked to quantify alcohol consumption pre-pregnancy in terms of the number and type of drinks consumed in a typical week using a laminated drinks card indicating different types of alcoholic drinks including brand names. They were asked about the frequency of binge drinking episodes (defined as more than five units of alcohol in one sitting) in a typical week. The same questions were asked in relation to a typical week in the first trimester of pregnancy and again in the third trimester questionnaire. Women who reported never drinking alcohol or who abstained entirely throughout pregnancy were termed “non-drinkers”. Women who abstained from alcohol entirely in the first trimester and who drank any alcohol in the third trimester were allocated to the “third trimester” group and women who reported any alcohol consumption in the first and third trimesters of pregnancy were allocated to the “first and third trimester” group. Two women reported alcohol intake in the first trimester but not the third trimester. However, the responses conflicted between the interview, reporting early pregnancy alcohol exposure, and the computerized data reporting only pre-pregnancy alcohol exposure. These women were included in the non-drinker category. A separate analysis of two women would not have allowed meaningful comparisons.

The data from the booking interview and third trimester questionnaire were linked to the electronic maternal and neonatal records with information on the mother and infant up until first hospital discharge. The medical records were reviewed for additional detailed information. Information on the following maternal characteristics was extracted from the electronic records: maternal age, marital status, socioeconomic group, nationality, public or privately funded antenatal care, parity, planned pregnancy, gestation at booking, smoking, alcohol use, illicit drug use, and referral to a social worker [11].

### 2.3. Antenatal Care

Each woman had a detailed booking interview in private with a midwife at the first antenatal visit. Women received routine advice on alcohol, smoking, diet, exercise and infant feeding. They were advised to abstain from alcohol, eat a healthy balanced diet, exercise regularly and breastfeeding was promoted. Women who continued to smoke were referred to support services for quitting smoking. Every woman had an ultrasound scan at the first antenatal visit and a further detailed structural anatomy scan at 20–22 weeks gestation. Gestational age was estimated from the calculation based on first day of the last menstrual period (adjusted for cycle length) but the booking ultrasound scan estimate was preferred if the dates were uncertain, the cycle was irregular or there was a discrepancy of more than seven days.

### 2.4. Perinatal Outcomes

Perinatal outcome measures included gestational age at delivery, live birth or stillbirth, birth weight, infant gender, infant’s condition at birth including Apgar scores at 1 and 5 minutes, admission to the neonatal unit, any suspected congenital abnormalities and whether resuscitation was required. Intrauterine growth restriction (IUGR) was defined as a birth weight less than the 10th percentile using individualised birth rate ratios (corrected for maternal height and weight, parity, infant gender, ethnicity and gestation, www.gestation.net).

### 2.5. Analysis

**Figure 1:**
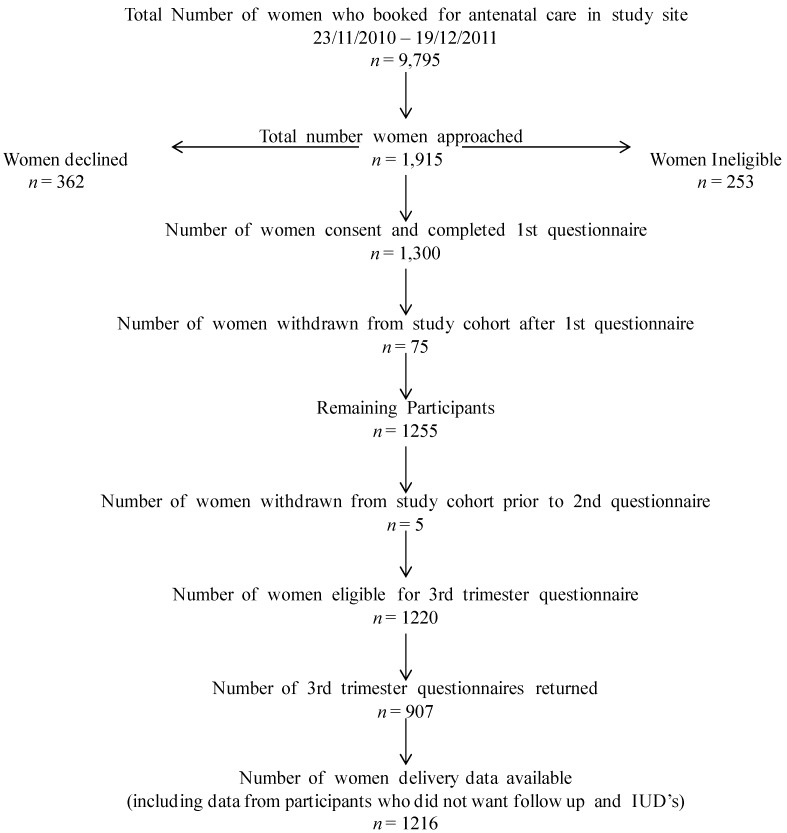
Cohort Flow Chart.

In total 1,915 women were approached of whom 1,300 agreed to participate in the study (Figure 1). Of these 1,216 delivered in the hospital and 907 (75%) completed the third trimester questionnaire., Participants were withdrawn from the cohort for a variety of reasons including miscarriage, molar pregnancy, multiple pregnancy, or a stated preference not to receive the third trimester questionnaire. The analyses were limited to the 907 mother-infant pairs on whom data was available from pre-pregnancy through to delivery. The analyses were performed using the Statistical Package for Social Sciences (SPSS version 20, IBM, Armonk, NY, USA). Descriptive statistics were used to characterise the study subjects by category of alcohol use. Comparisons were made between the three groups to identify socio-demographic factors associated with abstaining from alcohol in the first trimester only, or continuing to drink in the first and third trimesters of pregnancy. Logistic regression analyses were performed to measure the association between alcohol exposure and adverse perinatal outcomes. The “non-drinker” category was chosen as the comparator for each set of analyses as this was unlikely to be biased by under-reporting. Logistic regression analyses were performed adjusting for potential confounding factors including maternal age, nationality, private healthcare, smoking, and history of illicit drug use. These factors were chosen because of their known or possible association with adverse perinatal outcome and because of baseline differences between the groups. Results are reported as proportions, crude odds ratios (OR) and adjusted odds ratios (OR) with 95% confidence intervals (CI). All of the chosen variables for the logistic regression models are required data items on the computer system, therefore we had very little missing data.

The study received the approval of the Coombe Women and Infants University Hospital’s research ethics committee: Study No. 22-2009.

## 3. Results

### 3.1. Descriptive Statistics

The socio-demographic characteristics of the cohort were similar at the time of recruitment, in the third trimester and at delivery (Table 1). Similarly, the study cohort was comparable to the general hospital population [12,13] although there was a higher proportion of non-Irish participants and lower proportion of private patients. This reflected more efficient rates of recruitment in the public clinics where higher numbers of women booked for care. The median gestational age at booking was 12.3 weeks.

In the six months prior to pregnancy 731 (81%) women reported consuming alcohol and 176 (19%) were non-drinkers. At the booking interview 441 (60%) of the prior drinkers had ceased drinking and abstained for the remainder of the pregnancy, 185 (25%) had ceased drinking but consumed alcohol in the third trimester, and 110 (15%) continued to drink in the first and third trimesters of pregnancy. Awareness of the units of alcohol in alcoholic drinks and of the recommended alcohol units women should not exceed was low overall but higher among women who consumed alcohol throughout pregnancy. (Table 2) Alcohol consumption pre-pregnancy was far higher than alcohol consumption during pregnancy, as was the frequency of binge drinking (*p* < 0.05). Women who continued to drink throughout pregnancy were more likely to report heavy alcohol intake pre-pregnancy than women who abstained from alcohol in pregnancy (23% consumed ≥15 units per week *vs*. 13%, *p* < 0.05). Wine and beer were more commonly consumed although 16% of women who consumed alcohol throughout pregnancy drank spirits. The majority of women (92%) who consumed alcohol in the first trimester drank five units or less. In the third trimester most women (72–75%) consumed five units of alcohol or less, but the number of women drinking 6–14 units increased three fold.

**Table 1:** Characteristics of Study Cohort in relation to the hospital population.

Characteristic	Study Population at Recruitment ^i^ *n* = 1,300 (%)	Study Population at Third Trimester ^ii^ *n* = 907 (%)	Study Population at Delivery ^iii^ *n* = 1,216 (%)	General Hospital Population ^iv^ *n* = 6,720 (%)
Maternal age at booking				
<20 years	34 (2.6)	19 (2.1)	31 (2.5)	200 (3.0)
20–24 years	161 (12.4)	102 (11.2)	152 (12.5)	776 (11.6)
25–29 years	362 (27.8)	235 (25.9)	336 (27.6)	1,527 (22.7)
30–34 years	453 (34.8)	334 (36.8)	427 (35.1)	2,322 (34.6)
35–39 years	247 (19.0)	188 (20.7)	232 (19.1)	1,592 (23.7)
>40 years	43 (3.3)	29 (3.2)	38 (3.1)	301 (4.5)
Marital status				
Married	679 (52.2)	505 (55.7)	635 (52.2)	3,952 (58.5)
Single	621 (47.8)	402 (44.3)	581 (47.8)	2,685 (40.0)
Socioeconomic group				
Professional	341 (26.2)	258 (28.4)	317 (26.1)	2,077 (30.9)
Home duties	222 (17.1)	135 (14.9)	206 (16.9)	961 (14.3)
Non-manual	491 (37.8)	369 (40.7)	481 (39.6)	2,622 (39.0)
Manual	65 (5.0)	44 (4.9)	46 (3.8)	267 (4.0)
Unemployed	117 (9.0)	50 (5.5)	103 (8.5)	501 (7.5)
Non-classifiable	64 (4.9)	51 (5.6)	63 (5.2)	289 (4.3)
Nationality				
Irish	888 (68.3)	618 (68.1)	839 (69.0)	5,510 (82.0)
Non-Irish	412 (31.7)	289 (31.9)	377 (31.0)	1,189 (17.7)
Gestation at booking *				
<12 weeks	528 (40.8)	369 (40.7)	493 (40.5)	2,666 (39.8)
12–20 weeks	729 (56.3)	523 (57.7)	687 (56.5)	3,683 (55.0)
>20 weeks	37 (2.9)	15 (1.7)	36 (3.0)	349 (5.2)
Private Health Care				
Yes	145 (11.2)	122 (13.5)	142 (11.7)	1,219 (18.1)
No	1,155 (88.8)	785 (86.5)	1,074 (88.3)	5,499 (81.9)

^i^ Recruitment took place at participants first antenatal visit to the hospital (usually 10–14 weeks’); ^ii^ The third trimester questionnaire was completed by participants from 28 weeks’ gestation; ^iii^ Study population at delivery includes intrauterine death *n* = 7 and neonatal death *n* = 1; ^iv^ General hospital population—Murphy *et al.* (2013) [13]; * Missing data for gestational age at booking *n* = 6.

**Table 2:** Alcohol-related knowledge and behaviours according to alcohol exposure in pregnancy.

Total *n* = 731 ^i^	Ex-Drinker All Pregnancy *n* = 441 (%)	Third Trimester Drinker Only *n* = 185 (%)	First and Third Trimester Drinker *n* = 105 (%)
Aware of units of alcohol in drinks(moderate or good knowledge)	46 (10.4)	21 (11.3)	20 (19.1) *p* = 0.03
Aware of recommended alcohol units women should not exceed	35 (7.9)	16 (8.6)	16 (15.2) *p* = 0.02
Units consumed pre-pregnancy <1–5 (per week) 6–14 15–20 >20 Any binge drinking episode	279 (63.3) 127 (28.8) 24 (5.4) 33 (7.5) 174 (39.5)	73 (39.5) 72 (38.9) 23 (12.4) 17 (9.2) 86 (46.5)	44 (41.9) 37 (35.3) 9 (8.6) 15 (14.3) *p* = 0.01 56 (53.3) *p* = 0.03
Alcohol source ^ii^ Alcopop Beer Wine Cider Spirits	30 (6.8) 102 (23.1) 154 (34.9) 21 (4.8) 51 (11.6)	7 (3.7) 58 (31.4) 90 (48.7) 13 (7.0) 19 (10.3)	6 (5.8) 33 (31.4) 50 (47.6) 8 (7.6) 17 (16.2)
Units consumed first trimester <1–5 (per week) 6–14 15–20 >20 Any binge drinking episode	__ __ __ __ __	__ __ __ __ __	97 (92.4) 7 (6.7) ∫ 1 (1.0) 0 (0.0) 1 (1.0)
Units consumed third trimester <1–5 (per week) 6–14 15–20 >20 Any binge drinking episode	__ __ __ __ __	135 (73.0) 30 (16.3) 3 (1.6) 0 (0.0) 5 (2.7)	79 (75.2) 21 (20.0) ∫ 1 (1.0) 2 (2.0) 7 (6.7)

^i^ Excludes never drinkers, *n* = 176; ^ii^ Some women consume more than one type of alcoholic drink; * Chi-square test for difference in proportions ∫ *p* < 0.01.

The characteristics of the women in the cohort in relation to alcohol in pregnancy are presented in Table 3. Compared to non-drinkers, third trimester drinkers were more likely to be older, OR 1.74 (95% CI 1.22, 2.49) for age 30–39 years, Irish OR 2.44 (95% CI 1.65, 3.63), to have a professional occupation OR 1.73 (95% CI 1.01, 2.97), private healthcare OR 1.75 (95% CI 1.11, 2.76) and to have a history of illicit drug use OR 2.21 (95% CI 1.39, 3.51). Continuing alcohol consumption during the first and third trimesters was associated with older maternal age OR 2.24 (95% CI 1.39, 3.60) for age 30–39 years, Irish Nationality OR 2.95 (95% CI 1.73, 5.03), private healthcare OR 2.02 (95% CI 1.18, 3.47), smoking OR 3.82 (95% CI 2.30, 6.36) and a history of illicit drug use OR 2.91 (95% CI 1.71, 4.95).

**Table 3:** Characteristics of women according to alcohol exposure in pregnancy.

Total *n* = 907	Non-Drinker *n* = 617 (%)	Third trimester only *n* = 185 (%)	First and Third Trimester *n* = 105 (%)	Odds ratio ^i^ 95% Confidence Intervals	Odds ratio ^ii^ 95% Confidence Intervals
Maternal age < 20 years 20–29 years ^∫^ 30–39 years >40 years	16 (2.6) 256 (41.5) 326 (52.8) 19 (3.1)	2 (1.1) 55 (29.7) 122 (66.0) 6 (3.2)	1 (1.0)26 (24.7)74 (70.5)4 (3.8)	0.58 (0.13–2.60) 1.00 1.74 (1.22–2.49) * 1.47 (0.56–3.85)	0.62 (0.08–4.83) 1.00 2.24 (1.39–3.60) *2.07 (0.66–6.55)
Single Marital status	260 (42.1)	90 (48.6)	52 (49.5)	1.30 (0.94–1.81)	1.35 (0.89–2.04)
Socioeconomic group Professional Home duties ^∫^ Non-manual Manual Unemployed Non-classifiable	159 (25.8) 99 (16.0) 262 (42.5) 31 (5.0) 31 (5.0) 35 (5.7)	64 (34.6) 23 (12.4) 73 (39.5) 7 (3.8) 12 (6.5) 6 (3.2)	35 (33.3) 13 (12.4) 34 (32.4) 6 (5.7) 7 (6.7) 10 (9.5)	1.73 (1.01–2.97) *1.00 1.20 (0.71–2.02) 0.97 (0.38–2.48) 1.67 (0.74–3.73) 0.74 (0.28–1.96)	1.68 (0.85–3.32)1.00 0.99 (0.50–1.95) 1.47 (0.52–4.20) 1.72 (0.63–4.69) 2.18 (0.88-5.41)
Irish Nationality	383 (62.1)	148 (80.0)	87 (82.9)	2.44 (1.65–3.63) *	2.95 (1.73–5.03) *
Private Health Care	68 (11.0)	33 (17.8)	21 (20.0)	1.75 (1.11–2.76) *	2.02 (1.18–3.47) *
Nulliparous	290 (47.0)	91 (49.2)	38 (36.2)	1.09 (0.79–1.52)	0.64 (0.42–0.98)
Unplanned pregnancy	202 (32.7)	52 (28.1)	34 (32.4)	0.80 (0.56–1.15)	0.98 (0.63–1.53)
Gestation at booking <12 weeks ^∫^ 12–20 weeks >20 weeks	255 (41.3) 351 (56.9) 11 (1.8)	78 (42.2) 105 (56.8) 2 (1.1)	36 (34.3) 67 (63.8) 2 (1.9)	1.00 0.98 (0.70–1.37) 0.59 (0.13–2.74)	1.00 1.35 (0.87–2.09) 1.29 (0.27–6.05)
Current smoker	56 (9.1)	25 (13.5)	29 (27.6)	1.57 (0.95–2.59)	3.82 (2.30–6.36) *
Illicit drug use (ever)	57 (9.2)	34 (18.4)	24 (22.9)	2.21 (1.39–3.51) *	2.91 (1.71–4.95) *
Social worker referral	16 (2.6)	5 (2.7)	1 (1.0)	1.04 (0.38–2.75)	0.36 (0.05–2.75)

^i^ Third trimester only *vs*. Non-drinker; ^ii^ First and Third trimester *vs*. Non-drinker; ^∫^ Reference category; * *p* < 0.05; Missing data for gestational age *n* = 6.

### 3.2. Perinatal Outcomes

Women who consumed alcohol in the first and third trimesters of pregnancy had very similar perinatal outcomes to non-drinkers (Table 4). There were no significant differences in preterm birth, low birth weight and IUGR between women who consumed alcohol in pregnancy and non-drinkers. The odds ratios for IUGR in particular, were attenuated after controlling for smoking.

There was no evidence of a step-wise increase in adverse perinatal outcomes with increasing alcohol exposure through the first and third trimesters.

**Table 4:** Perinatal outcomes according to alcohol exposure in first and third trimester of pregnancy and third trimester only.

Alcohol Intake	Non-Drinker *n* = 617	Third Trimester Only Alcohol *n* = 185	First and Third Trimester Alcohol *n* = 105	Third Trimester Only *vs*. Non-Drinker Odds Ratio (95% CI) Adjusted ^i^ OR (95% CI)	First and Third Trimester *vs*. Non-Drinker Odds Ratio (95% CI) Adjusted ^i^ OR (95% CI)
Gestational age (weeks) Mean (SD) Range Mean difference (95% CI)	39.6 (1.5) 29–42	40.0 (1.5) 32–42	39.7 (1.5) 32–42	0.4 (0.2–0.6)*	0.1 (−0.2–0.4)
Birth weight (g) Mean (SD) Range Mean difference (95% CI)	3,460 (498) 1,145–5,160	3,494 (500) 1,970–5,030	3,527 (571) 1,530–4,700	34 (−48–116)	67 (−39–173)
Preterm birth <37 weeks (%)	33 (5.3)	6 (3.2)	3 (2.9)	0.77 (0.50–1.20) 0.78 (0.50–1.37)	0.52 (0.16–1.73) 0.50 (0.15–1.74)
Low birth weight <2,500 g (%)	23 (3.7)	5 (2.7)	3 (2.9)	0.72 (0.27–1.91) 0.83 (0.50–1.37)	0.76 (0.22–2.58) 0.67 (0.19–2.39)
Intrauterine growth restriction ^i, ii ^(%)	80 (13.0)	32 (17.3)	13 (12.4)	1.40 (0.90–2.20) 1.22 (0.97–1.55)	0.95 (0.51–1.77) 0.90 (0.46–1.77)
Apgar score <7 at 5 min (%)	5 (0.9)	1 (0.5)	1 (1.0)	0.67 (0.08–5.73) 1.00 (0.34–3.04)	1.18 (0.14–10.2) 1.30 (0.13–12.68)
Admitted to neonatal unit (%)	108 (17.5)	29 (15.7)	11 (10.5)	0.88 (0.56–1.37) 0.91 (0.71–1.16)	0.55 (0.29–1.07) 0.46 (0.22–1.01)

^i^ Adjusted for maternal age, socio-economic group, Irish nationality, private healthcare, current smoker, history of illicit drug use; ^ii^ Customised birth weight <10th percentile; * *p* < 0.05.

## 4. Discussion

### 4.1. Summary of Main Findings

This study found that almost 60% of prior drinkers attending for antenatal care had made a decision to abstain from alcohol by the time of the first antenatal visit and remained abstinent throughout pregnancy. A further 25% abstained in the first trimester and resumed drinking in the third trimester, and 15% consumed alcohol in both trimesters. Although few women reported an awareness of alcohol units and recommendations, many women appeared to avoid alcohol in the first trimester but relax their approach both in terms of exposure and units consumed when they reached the third trimester. Despite this we found no evidence of adverse perinatal outcomes in association with either exposure in the third trimester only when fetal growth is maximal, or during the first and third trimesters when both development and growth occur.

### 4.2. Strengths and Limitations of the Study

The study cohort was representative of women attending a large university teaching hospital between 2010 and 2011. We had detailed information on lifestyle behaviours and ascertained data three different ways at two separate time points. The potential for recall bias was limited. The data on alcohol consumption relied on self-reporting by the pregnant woman and it is possible that alcohol exposure was under-reported [14]. There was a loss of responders in the third trimester despite written reminders and telephone contact, however the profile of the cohort at the different time points suggests that the loss to follow-up was random. As with any study of this type, it was not feasible to recruit all women booking for antenatal care at the hospital. It is possible that the behaviours and outcomes of those who decline to participate in research differ from those who do.

### 4.3. Comparison with Existing Literature

In keeping with our study, Henderson *et al.* found no consistent evidence of adverse effects of low to moderate prenatal alcohol exposure on pregnancy outcome [7]. However, a meta-analysis of the dose-response relationship between alcohol consumption before and during pregnancy indicated that heavy alcohol consumption was associated with an increased risk of low birth weight, preterm birth and small for gestational age (SGA) [6]. In contrast a recent cohort study reported that for healthy women having their first baby, there was no association between alcohol consumption before 15 weeks of gestation and small for gestational age, reduced birth weight or spontaneous preterm birth, even at higher levels of consumption or with binge drinking [15].

Like our study, a Swedish study demonstrated that although women reported continued alcohol use during pregnancy, the biomarkers indicated only modest drinking levels, and this may explain a lack of association with adverse perinatal outcomes [16]. Similarly, a prospective cohort study of light and moderate maternal alcohol consumption in The Netherlands showed no consistent associations between the number of alcoholic drinks consumed and fetal growth patterns [17]. Given that most women moderated their alcohol consumption considerably it is likely that our study was under-powered to detect associations between alcohol exposure and adverse perinatal outcomes. However, the same cohort revealed strong associations between smoking and intrauterine growth restriction [13], and if anything the direction of associations for preterm birth and low birth weight in this cohort favoured alcohol exposure. This may represent a true effect, a type 1 error or residual confounding, and warrants further research.

Similar to our study, others have reported very positive changes in pregnancy in relation to lifestyle behaviours and this appears to be largely self-motivated [2]. Unlike most other studies, we performed a longitudinal study of alcohol consumption pre-pregnancy, in the first trimester and in the third trimester, recruiting from an unselected population of both nulliparous and parous women, which gives new perspectives on lifestyle behaviours in pregnancy.

### 4.4. Implications for Practice

There is a culture of high alcohol consumption in North European countries that includes women of reproductive age. This has implications for both unplanned pregnancy and unintended excess peri-conceptional alcohol exposure [3]. This study demonstrates that there is very limited awareness among pregnant women of alcohol units in drinks consumed, and of the upper limit of alcohol units recommended for women. Despite this, the majority of women moderate their behaviour in the first trimester of pregnancy, probably reflecting a basic understanding of human biology and fetal development. What is perhaps surprising is that continued drinking in pregnancy is associated with older maternal age and higher educational attainment, a finding that has been reported in other settings [2,15]. Public health campaigns need to address this gap in both understanding and behaviour modification.

An additional concern is the relaxation in behaviour in the third trimester with more women drinking alcohol, and those who drink, drinking more. Currently, health promotion initiatives in pregnancy are focussed on the first antenatal booking visit, where there are many competing issues to discuss [11]. Parent education classes in the third trimester provide an ideal opportunity to reinforce the importance of healthy lifestyle behaviours. Although there was no evidence in this study of adverse perinatal outcomes in association with alcohol consumption in the first and third trimesters of pregnancy, this relationship is complex with confounding factors working in different directions. In particular, the u-shaped association between alcohol consumption and social advantage/disadvantage warrants further attention. It is also important to emphasise that this study provides no reassurances about subsequent childhood development. Given current advice from the RCOG, that there are no known health benefits of drinking alcohol in pregnancy, the safest approach remains one of abstinence [18].

## 5. Conclusions

Despite limited awareness of alcohol units and recommendations, most women modify their alcohol intake to a low level in pregnancy. Although there was no evidence of adverse perinatal outcomes in association with low level alcohol consumption in the first and third trimesters of pregnancy, the safest approach remains one of abstinence.

## Appendix

STROBE Statement—Checklist of items that should be included in reports of *cohort studies*

**Table:**

Item	Item No	Recommendation
Title and abstract	1	(*a*) Indicate the study’s design with a commonly used term in the title or the abstract **Included—title and abstract**
		(*b*) Provide in the abstract an informative and balanced summary of what was done and what was found **Included, page 2**
**Introduction**		
Background/Rationale	2	Explain the scientific background and rationale for the investigation being reported **Included, page 4–5**
Objectives	3	State specific objectives, including any prespecified hypotheses **Included, page 5**
**Methods**		
Study design	4	Present key elements of study design early in the paper **Included, page 5–7**
Setting	5	Describe the setting, locations, and relevant dates, including periods of recruitment, exposure, follow-up, and data collection **Included, Page 5**
Participants	6	(*a*) Give the eligibility criteria, and the sources and methods of selection of participants. Describe methods of follow-up **Included, page 5–6**
		(*b*)For matched studies, give matching criteria and number of exposed and unexposed **Not applicable**
Variables	7	Clearly define all outcomes, exposures, predictors, potential confounders, and effect modifiers. Give diagnostic criteria, if applicable **Included, page 5–7**
Data sources/Measurement	8 *	For each variable of interest, give sources of data and details of methods of assessment (measurement). Describe comparability of assessment methods if there is more than one group **Included, page 5–7**
Bias	9	Describe any efforts to address potential sources of bias **Not applicable, complete cohort**
Study size	10	Explain how the study size was arrived at **Time limited cohort**
Quantitative variables	11	Explain how quantitative variables were handled in the analyses. If applicable, describe which groupings were chosen and why **Described statistics section**
Statistical methods	12	(a) Describe all statistical methods, including those used to control for confounding
		(b) Describe any methods used to examine subgroups and interactions
		(c) Explain how missing data were addressed
		(d) If applicable, explain how loss to follow-up was addressed
		(e) Describe any sensitivity analyses **Described, statistics section**
**Results**		
Participants	13 *****	(a) Report numbers of individuals at each stage of study—e.g., numbers potentially eligible, examined for eligibility, confirmed eligible, included in the study, completing follow-up, and analysed **See footnote Table 1and methods section**
		(b) Give reasons for non-participation at each stage
		(c) Consider use of a flow diagram
Descriptive data	14 *****	(a) Give characteristics of study participants (e.g., demographic, clinical, social) and information on exposures and potential confounders
		(b) Indicate number of participants with missing data for each variable of interest
		(c) Summarise follow-up time (e.g., average and total amount) **Descriptive data presented - results and Tables**
Outcome data	15 *****	Report numbers of outcome events or summary measures over time **Outcome data presented - results and Tables**
Main results	16	(a) Give unadjusted estimates and, if applicable, confounder-adjusted estimates and their precision (e.g., 95% confidence interval). Make clear which confounders were adjusted for and why they were included
		(b) Report category boundaries when continuous variables were categorized
		(c) If relevant, consider translating estimates of relative risk into absolute risk for a meaningful time period **Appropriate estimates adjusted—unadjusted presented**
Other analyses	17	Report other analyses done—e.g., analyses of subgroups and interactions, and sensitivity analyses
**Discussion**		
Key results	18	Summarise key results with reference to study objectives
Limitations	19	Discuss limitations of the study, taking into account sources of potential bias or imprecision. Discuss both direction and magnitude of any potential bias
Interpretation	20	Give a cautious overall interpretation of results considering objectives, limitations, multiplicity of analyses, results from similar studies, and other relevant evidence
Generalisability	21	Discuss the generalisability (external validity) of the study results **Discussion follows recommended format**
**Other information**		
Funding	22	Give the source of funding and the role of the funders for the present study and, if applicable, for the original study on which the present article is based **Included, page 14**

***** Give information separately for exposed and unexposed groups. **Note:** An Explanation and Elaboration article discusses each checklist item and gives methodological background and published examples of transparent reporting. The STROBE checklist is best used in conjunction with this article (freely available on the Web sites of PLoS Medicine at http://www.plosmedicine.org/, Annals of Internal Medicine at http://www.annals.org/, and Epidemiology at http://www.epidem.com). Information on the STROBE Initiative is available at http://www.strobe-statement.org.
